# Effect of Solar Particle Event Radiation and Hindlimb Suspension on Gastrointestinal Tract Bacterial Translocation and Immune Activation

**DOI:** 10.1371/journal.pone.0044329

**Published:** 2012-09-19

**Authors:** Yu Zhou, Houping Ni, Minghong Li, Jenine K. Sanzari, Eric S. Diffenderfer, Liyong Lin, Ann R. Kennedy, Drew Weissman

**Affiliations:** 1 Division of Infectious Diseases, Department of Medicine, Perelman School of Medicine, University of Pennsylvania, Philadelphia, Pennsylvania, United States of America; 2 Department of Radiation Oncology, Perelman School of Medicine, University of Pennsylvania, Philadelphia, Pennsylvania, United States of America; Rush University, United States of America

## Abstract

The environmental conditions that could lead to an increased risk for the development of an infection during prolonged space flight include: microgravity, stress, radiation, disturbance of circadian rhythms, and altered nutritional intake. A large body of literature exists on the impairment of the immune system by space flight. With the advent of missions outside the Earth's magnetic field, the increased risk of adverse effects due to exposure to radiation from a solar particle event (SPE) needs to be considered. Using models of reduced gravity and SPE radiation, we identify that either 2 Gy of radiation or hindlimb suspension alone leads to activation of the innate immune system and the two together are synergistic. The mechanism for the transient systemic immune activation is a reduced ability of the GI tract to contain bacterial products. The identification of mechanisms responsible for immune dysfunction during extended space missions will allow the development of specific countermeasures.

## Introduction

Future space missions will involve distant travel and extended stays outside the Earth's magnetic field that provides protection from solar radiation. Multiple environmental factors have been identified that increase the risk of infection during these missions that include; stress [1], reduced weight bearing (reviewed in [2]), disturbance of circadian rhythms [3], and altered nutritional intake [4], in addition to solar and galactic radiation [5], [6]. These factors, either alone, independently additive, or through synergistic interactions, pose a threat for the development of pathogenic infection by exogenous or endogenous organisms [7], [8]. Exogenous organisms are present in other astronauts or the spacecraft and endogenous organisms, which are resident in the astronaut at the start of space flight, consist of latent viruses common in humans (e.g., Epstein-Barr, Herpes simplex, cytomegalovirus, and others) or commensal and colonizing pathogenic organisms [5], [9], [10], [11]. In addition to the effects on the host immune system, space flight has also been shown to decrease antibiotic potency and enhance microbial virulence [12]. The consistent effects on the immune system observed during space travel, thus far, are a decrease in NK cell number and functionality [13], [14], decreases in cell-mediated immunity with altered cytokine production [14], [15], and no decrease in levels of serum immunoglobulins [14].

A large body of literature exists on the impairment of the immune system in space flight models, namely versions of hindlimb unloading, demonstrating suppression of bone marrow function and altered innate and acquired immunity (reviewed in [16], [17]). These models have also demonstrated a reduced ability to clear infections by *Klebsiella pneumonia* [18] and *Pseudomonas aeruginosa* [19]. Many similarities have been found in comparing hindlimb unloading models to animals flown in space, including a reduction in thymic volume [20], [21], decreased IFN-γ production [22], [23], and reduced lymphocyte blastogenesis [24], [25], [26], as well as a number of differences, including leukocyte subset alterations (reviewed in [27]).

Interplanetary radiation is primarily composed of galactic cosmic rays and solar radiation [28], which consists of low energy solar wind and more energetic solar particle events (SPEs) that originate from magnetically disturbed regions of the Sun [29], [30]. SPEs cannot be predicted and they typically can last for several hours, although some may continue for several days. They are composed predominately of low energy protons with helium ions (∼10%) and heavy ions and electrons (∼1%). SPEs occur predominantly around the time of solar maximum, but large events are more likely to occur in the ascending or declining phases of a solar cycle. This sporadic behavior complicates mission planning. During the past 5 complete solar cycles, between 0 and 5 (average of 2.6) large SPEs, with energies above 30 MeV and exceeding 10^9^ protons/cm^2^, which are levels that could affect an astronaut, were recorded (NASA publication, Human Health and Performance Risks of Space Exploration Missions). The confines of a space capsule or permanent enclosure will protect astronauts from SPE radiation, but astronauts are at risk of exposure when performing extra-vehicular activities. It is predicted that astronauts may receive up to 2 Gy of radiation to the bone marrow [31] and up to ∼32 Gy to the skin [32] during a strong SPE.

We previously demonstrated that SPE-like radiation at energies and dose rates observed in a strong SPE led to transient immune activation that was associated with an increase in circulating LPS [33]. Similar findings were observed with proton or gamma radiation and delivery at low or high dose rates. Two Gy of proton radiation, the maximum deep body dose expected during a strong SPE [31], led to transient increases in markers of immune activation and a measurable breakdown in the integrity of tight junctions of the GI tract columnar epithelial layer [33]. This study only analyzed a single space stressor, SPE radiation. In the current study, we have extended our observations to include most of the predicted major immune stresses expected during extended space travel outside of the Earth's magnetic field, including weightless conditions, altered nutritional intake, SPE-like radiation, and situational and confinement stress and found that the combination of these space travel related environmental conditions resulted in enhanced levels of systemic innate immune activation that had a significantly extended duration beyond the exposure to radiation.

## Materials and Methods 

### Ethics statement

This study was carried out in strict accordance with the recommendations in the Guide for the Care and Use of Laboratory Animals of the National Institutes of Health. The protocol was approved by the Institutional Animal Care and Use Committee of the University of Pennsylvania (#802238).

### Irradiation of mice

Female outbred ICR mice, 5–6 weeks of age, were obtained from Taconic Farms Inc. (Germantown, NY). For irradiation, the mice were placed in aerated plastic chambers (AMAC #530C). The chambers allowed the mice to easily turn around (reverse nose to tail direction). The mice were exposed to either gamma or proton radiation as follows. For the gamma radiation, mice were exposed to a total body dose of 0.5 to 2 Gy ^137^Cs gamma radiation, which was administered at a dose rate of 44 cGy/minute in a Gammacell 40 irradiator. The proton beam was produced by an IBA cyclotron system. The 230 MeV proton beam extracted from the cyclotron was degraded using the energy selection system to a nominal energy of 151 MeV or range of 16 cm water equivalent thickness (WET). The degraded beam was delivered in double scattering mode with a spread out Bragg peak modulation width of 5 cm. A 23 cm×17 cm opening in the tungsten multi-leaf collimator shaped the beam to a useable field size (>95% of maximum within the flat region) of 20.6 cm×17 cm at the gantry isocenter. Up to eight mice enclosures with dimensions of 7.2 cm×4.1 cm×4.1 cm were arranged in a 2×4 array forming a 14.2 cm×16.4 cm target area. The center of the enclosure array was placed at the gantry isocenter with an additional 11 cm WET of solid water plastic placed directly in front of the array further degrading the proton beam energy to approximately 78.4 MeV or a range of 5 cm WET. Five cm WET of solid water plastic was placed directly behind the enclosure array. Dosimetry verification was performed before the irradiations with a 2D ion chamber array (I'm*RT* MatriXX, IBA dosimetry) placed at a depth of 13.3 cm WET. The proton radiation exposures were delivered in a single fraction at a dose rate of 50 cGy/min. Non-irradiated mice were placed in the same chambers for the same amount of time.

### Hindlimb unloading

Mice were hindlimb unloaded as described previously [34]. Individual mice were suspended by the tail at 15° head-down tilt with no load bearing on the hindlimbs. Access to food and water was ensured using both water bottles and gel packs and food distributed around the floor of the cage. Animals demonstrated no adverse effects or pronounced weight loss. Groups of 5 mice per treatment per experiment were used due to hindlimb suspension cage limitations and each experiment was repeated at least 3 times resulting in a total of 15 or more mice in each measurement.

Blood was obtained at various times before and after hindlimb suspension and irradiation by cheek lancet. Serum was separated by centrifugation at 4,000 RPM for 4 min in an Eppendorf microfuge and frozen at −80°C. In certain experiments, animals were sacrificed 6 hr, 1, and 4 days after irradiation and tissues including terminal ileum were snap frozen on liquid nitrogen in OCT medium (Thermo Fisher) and kept at −80°C.

### Lipopolysaccharide (LPS) assay

Serum LPS was measured using an assay that utilizes the first component of the LAL reaction, Factor C (Lonza), with a sensitivity range of 0.01 to 10 EU/ml. Unlike the standard LAL assay that uses the hemolymph of horseshoe crabs (Limulus), which naturally clots in the presence of LPS, this assay uses the first component of the clotting cascade, Factor C, and a substrate that becomes fluorescent after cleavage. Serum was diluted 1 to 8 in endotoxin free water and heated to 80°C for 15 min, which reduced inhibitory activity as measured by spiking with LPS. Samples were run in duplicate.

### LPS binding protein (LBP), soluble (s)CD14, IL-6, TNF-α, and IFN-α ELISAs

Serum was analyzed for LBP, IFN-α, sCD14 (Cell Sciences, Canton, MA), IL-6 and TNF-α (R&D Systems, Minneapolis, MN) by direct binding ELISA, as described by the manufacturer. Serum was diluted 1 to 500 with PBS and analyzed in duplicate for LBP. Serum was diluted 1 to 100 with PBS for sCD14 and analyzed in duplicate. Serum was analyzed for IL-6, TNF-α, and IFN-α without dilution in duplicate.

### Immunohistochemistry

Two cm long pieces of terminal ileum just prior to the ascending colon were obtained from animals. Tissue was cut into 6 µm sections in a Leica CM1850 cryostat, fixed in acetone (Thermo Fisher), and stained with rabbit anti-mouse Claudin-3 IgG (Thermo Fisher), goat anti-Lipopolysaccharide IgG (US Biologicals), or a murine monoclonal antibody to Escherichia coli (J5) LPS (Acris Antibodies) and detected with biotinylated anti-rabbit, goat, or mouse IgG (Sigma), streptavidin horseradish-peroxidase (Vector Labs), and AEC substrate (Sigma). Tissue was counterstained with Hematoxylin (Electron Microscopy Sciences) and examined and photographed under brightfield microscopy (Nikon Eclipse e1000). Quantitation of breaks in Claudin-3 staining were made by counting the number of breaks per thousand enterocytes in tissue from 10 animals in each treatment group. Goblet cell counts were counted as a control.

### Statistics

Means, standard error of the mean, standard deviations, and ANOVA analyses with post-hoc group comparisons and Bonferroni correction were performed using StatPlus (AnalystSoft) and Microsoft Excel software.

## Results

**Figure 1:**
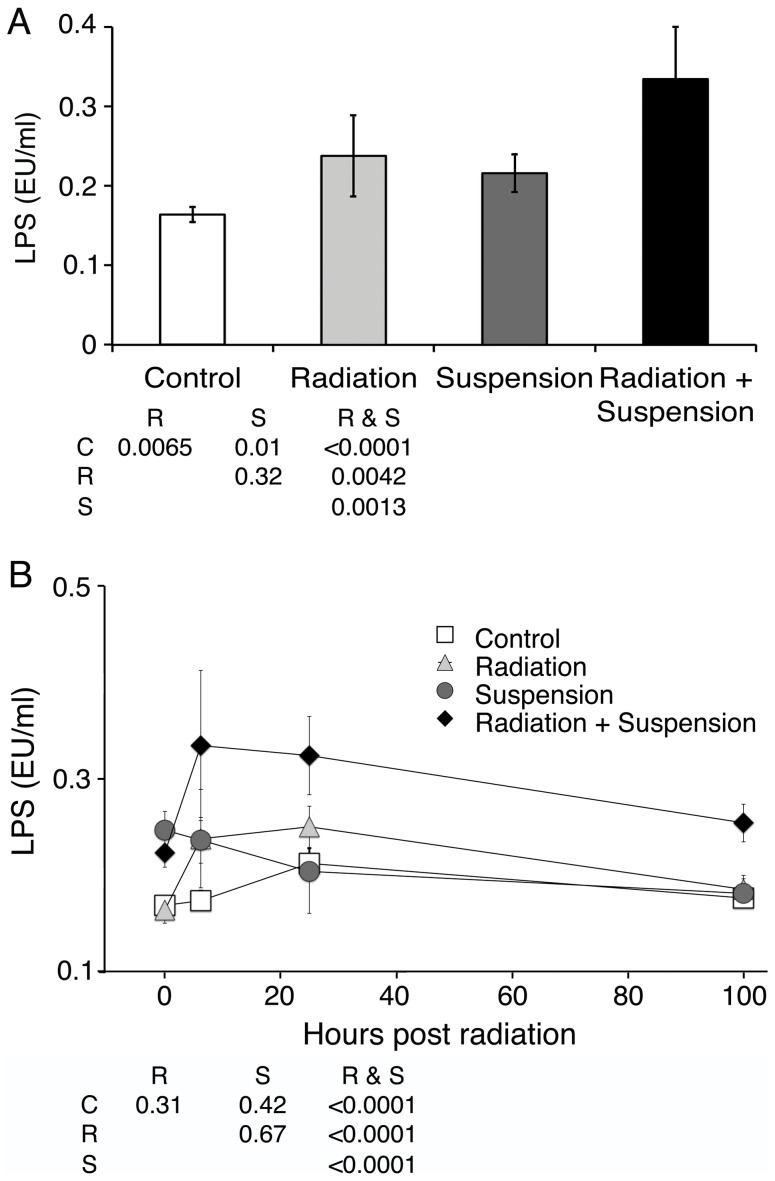
SPE-like proton or reference gamma radiation and hindlimb suspension increase circulating LPS levels. Hindlimb suspension and proton or gamma radiation cause a breakdown in containment of gram negative bacterial products as measured by circulating LPS. Mice were placed in hindlimb suspension or not and irradiated with 2 Gy of protons at 50 cGy/min (A) or 2 Gy of gamma radiation at 44 cGy/min (B) 2 days later or not. Six hours (A, B) after radiation and prior to and 1 and 4 days (B) post irradiation (2, 3, or 6 days post suspension), serum was analyzed for LPS content by Factor C assay. Data shown are the averages for five mice per group analyzed in duplicate; error bars are SEM. Statistically significant differences between groups is noted in the inset table (B is 4 days post) with C = control, R = radiation, S = suspension as calculated with ANOVA analyses with post-hoc group comparisons using Bonferroni correction. Data shown are from a single experiment and are representative of three experiments.

The main potential stresses on immune function during space travel include the weightless environment, altered nutritional intake, disturbances in circadian rhythm, situational and confinement stress, and exposure to radiation. The hindlimb unloading model, first used in 1985 (reviewed in [35], [36]) to model the weightless conditions of spaceflight, is the widely accepted land based model. In addition to mimicking weightless conditions, this model also induces situational and confinement stress and altered nutritional intake. To model the conditions that an astronaut would be exposed to SPE radiation, mice were suspended for 2 days and then irradiated with 2, 1, or 0.5 Gy of proton (70 MeV) or gamma radiation and placed back in suspension and analyzed over 7 days. Similar data was obtained when mice were suspended for 5 days prior to irradiation. Both irradiated and non-irradiated mice were placed in 7.2 cm×4.1 cm×4.1 cm enclosures for the same amount of time to control for the effects of this confinement. Certain groups received only suspension or radiation or were untreated. Mice were placed in similar caging with or without hindlimb suspension in the same room. Circulating levels of LPS were measured and demonstrated, as previously reported [33], 2 Gy of proton or gamma radiation led to a low but significant increase, 6 and 24 hr later (Figure 1A and B) compared to control animals (F = 21.9, p = 3×10^−8^ and F = 6.7, p = 0.01, respectively). Hindlimb unloading led to an increase in serum LPS at 6 hr (54 hr total suspension) and the combination of radiation and unloading demonstrated a larger increase (Figure 1A and B). At 4 days post irradiation, the circulating levels of LPS remained significantly elevated for animals that received both gamma radiation and suspension, while the other groups returned to baseline (Figure 1B) (F = 47.2, p = 1.8×10^−11^). Similar time course data were obtained with proton radiation. Lower doses of radiation, 1 and 0.5 Gy, did not significantly increase circulating levels of LPS either alone or with hindlimb suspension (Data not shown). We previously reported that live bacteria could not be cultured in tissue from 2 Gy irradiated mice [33]. We similarly did not find culturable bacteria in blood, spleen, kidneys, or lungs of irradiated and hindlimb suspended mice, but this does not exclude that live bacteria that do not grow in culture were not present.

**Figure 2:**
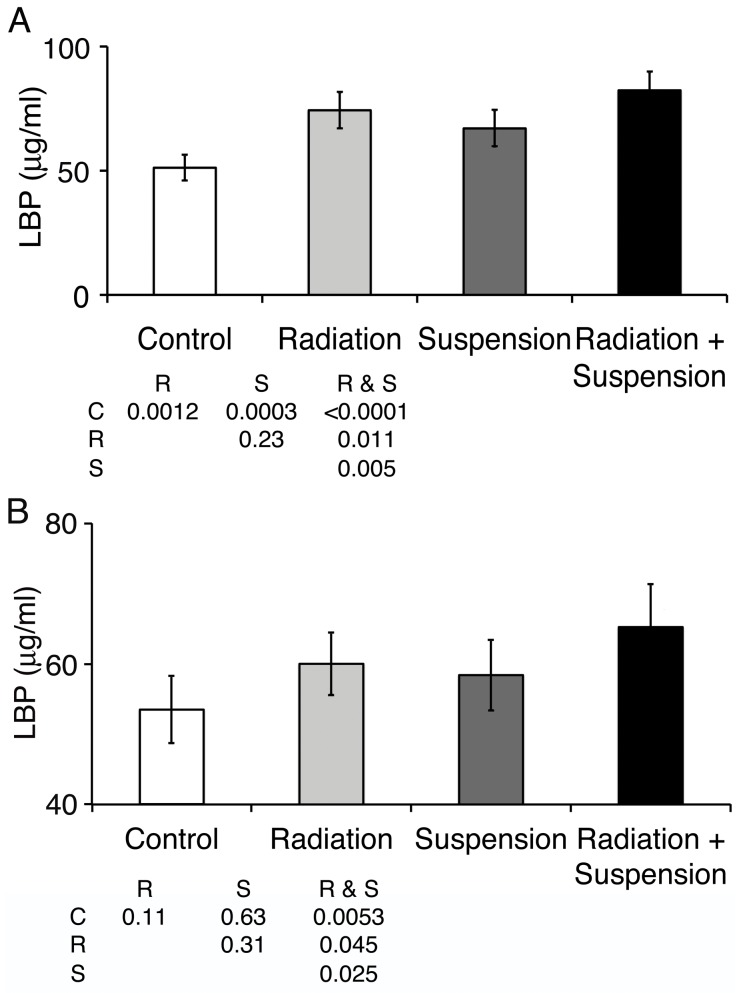
SPE-like radiation and hindlimb suspension increase the acute-phase reactant LPS binding protein. Mice were hindlimb suspended and/or irradiated with 2 Gy protons at 50 cGy/min. One (A) and 4 (B) days later, serum was obtained and analyzed for LPS binding protein content by ELISA. Data shown are the averages for five mice per group run in duplicate; error bars are SEM. Statistically significant differences between groups is noted in the inset table with C = control, R = radiation, S = suspension as calculated with ANOVA analyses with post-hoc group comparisons using Bonferroni correction. Data shown are from a single experiment representative of three experiments.

To determine whether the increase in circulating LPS induced a systemic response, the type I acute phase protein, LPS binding protein (LBP), was measured. LBP is a circulating protein that binds to LPS of Gram-negative bacteria via the lipid A region, which then promotes its binding to CD14 (reviewed in [37]). LBP is constitutively present and is induced during various types of infection and inflammatory processes. LBP was increased after radiation (proton) (24 hr) and hindlimb suspension (3 days total suspension) and was increased further when they were combined (Figure 2A) (F = 15.0, p = 1.7×10^−6^). At 4 days post radiation, or a total of 6 days of hindlimb suspension, LBP remained significantly elevated in the group that received both 2 Gy of radiation and suspension (Figure 2B) (F = 5.4, p = 0.014). Similar data was obtained with gamma radiation. This demonstrates that the increase in circulating LPS led to a systemic response and while radiation and suspension appeared additive on day 1 post radiation, the increase in LBP remained on day 4 only for the groups that received both 2 Gy of radiation and hindlimb unloading. Mice receiving 1 and 0.5 Gy of proton or gamma radiation and hindlimb suspension were similar to controls at day 4 (Data not shown).

**Figure 3:**
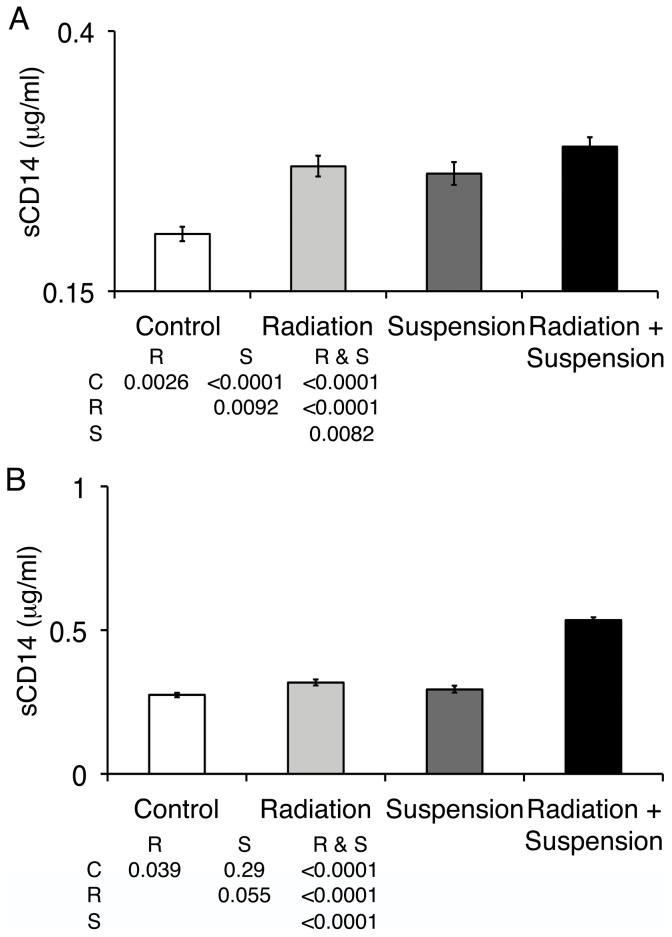
SPE-like or reference gamma radiation and hindlimb suspension increase sCD14 levels. Mice were hindlimb suspended and/or irradiated with 2 Gy of protons at 50 cGy/min (A) or gamma radiation at 44 cGy/min (B). One (A) and 4 (B) days later, serum was obtained and analyzed for sCD14 protein content by ELISA. Data shown are the averages for five mice per group run in duplicate; error bars are SEM. Statistically significant differences between groups is noted in the inset table with C = control, R = radiation, S = suspension as calculated with ANOVA analyses with post-hoc group comparisons using Bonferroni correction. Data shown are from a single experiment representative of three experiments.

LPS released from Gram-negative bacteria is an aggregate, due to its amphiphilic structure. LPS aggregates are transformed into monomers by LBP. LBP then delivers LPS monomers to CD14 [38], [39] that in turn delivers it to MD2-Toll-like receptor (TLR)4 resulting in signaling (reviewed in [40]). CD14 exists in 2 forms, a GPI-linked membrane protein and a soluble form made either by proteolytic release of the membrane form or secretion by hepatocytes (reviewed in [40]). LPS exposure leads to an increase in sCD14 through both of these mechanisms. sCD14 is a very sensitive marker of increased levels of circulating LPS [41]. The circulating levels of sCD14 24 hr after 2 Gy of proton or gamma radiation or 3 days after the initiation of suspension were significantly elevated and the combination of radiation and hindlimb unloading further increased sCD14 (Figure 3A and gamma data not shown) compared to control animals (F = 11.9, p = 0.00016). Four days after proton irradiation of suspended mice, the levels of sCD14 continued to be significantly elevated compared to control animals (F = 50.3, p = 8.2×10^−12^), while mice that received radiation or hindlimb unloading alone were not statistically different from control mice (Figure 3B and proton data not shown). Lower doses of radiation combined with hindlimb suspension did not result in elevated levels of circulating sCD14 four days after irradiation (Data not shown).

**Figure 4:**
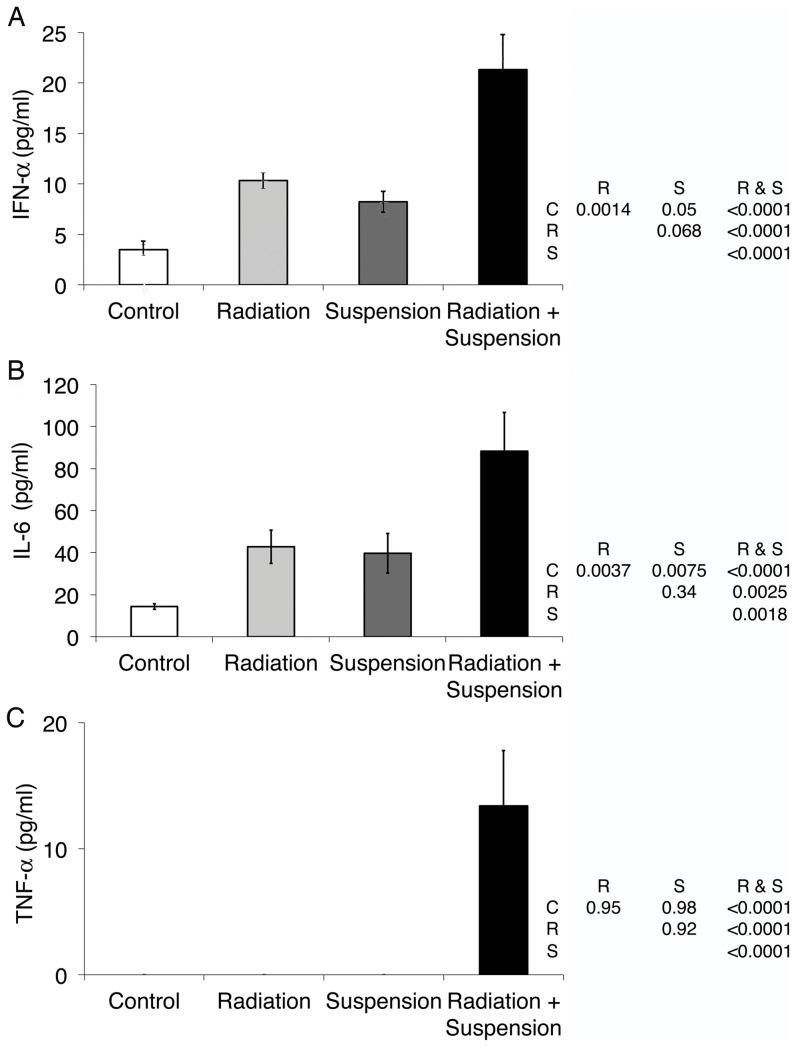
Radiation and hindlimb suspension induce a transient increase in circulating proinflammatory cytokines. Mice were irradiated with 2 Gy of gamma radiation at a dose rate of 44 cGy/min. Six hr later, serum was obtained and analyzed for IFN-α (A), IL-6 (B), and TNF-α (C) by ELISA. Data shown are the averages for 5 mice per group run in duplicate; error bars are SEM. Statistically significant differences between groups is noted in the inset table with C = control, R = radiation, S = suspension as calculated with ANOVA analyses with post-hoc group comparisons using Bonferroni correction. Data shown are from a single experiment representative of three experiments.

Bacterial translocation increases circulating levels of LPS, as well as other bacterial components [42], such as DNA [43], some of which can activate innate immune sensors. To measure whether the increase in bacterial product translocation induced by radiation and/or hindlimb unloading led to the induction of a systemic cytokine response, circulating levels of interferon (IFN)-α, TNF-α, and IL-6 were measured. Radiation (2 Gy of gamma or proton) and hindlimb unloading alone led to an increase in circulating IFN-α and IL-6 and at least additive levels were observed in mice treated with both (Figure 4 and proton data not shown) ((F = 7.9, p = 0.0019 and F = 15.2, p = 0.00018, respectively). The combination of irradiation and hindlimb unloading led to an increase in TNF-α (F = 6.8, p = 0.0045). Lower doses of radiation in combination with hindlimb suspension did not lead to significant increases in circulating TNF-α, IL-6, and IFN-α (Data not shown).

Circulating LPS in hindlimb unloaded and proton or gamma irradiated mice returned to baseline levels 7 days after exposure to radiation. Levels of circulating sCD14 and LBP also returned to control levels 7 days after irradiation with 2 Gy of proton or gamma radiation. The return to control levels occurred in the setting of continued hindlimb suspension, which suggested the impairments to containment of bacterial products decreased with continued time in suspension. This was evident in the analysis of circulating LPS, which indicated that at 3 days post initiation of hindlimb suspension, the levels of LPS decreased to baseline levels (Figure 1B). LBP and sCD14 levels returned to baseline by 4 days post irradiation and 6 days post suspension when either was administered alone (Figure 2B and 3B).

**Figure 5:**
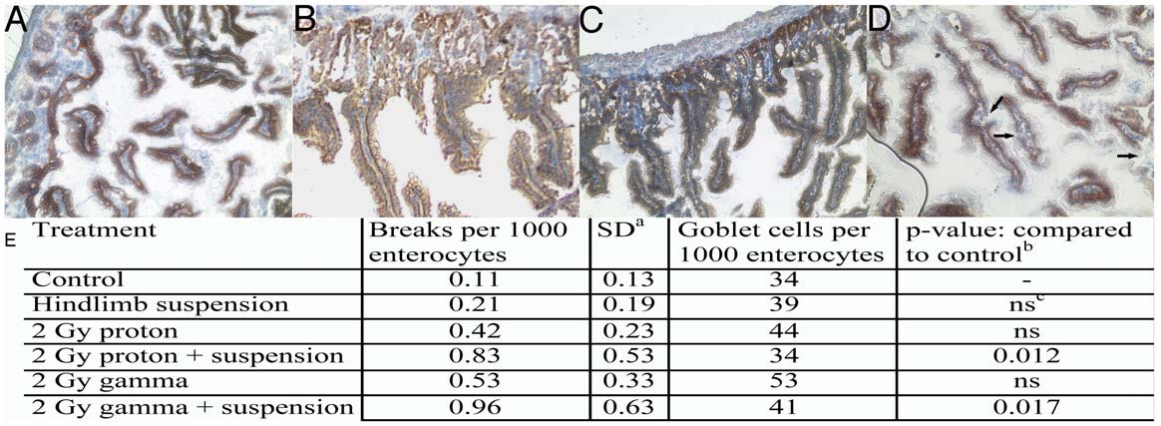
SPE-like radiation and hindlimb suspension induces breaks in the GI epithelial barrier. Terminal ileum obtained 4 days post irradiation and/or 6 days post hindlimb suspension or from control animals was stained for Claudin-3. A) Ileum from a control treated mouse. B) Ileum from a mouse irradiated with 2 Gy of 70 MeV protons. Black arrow indicates a region of tight junction incongruity. C) Ileum from a mouse subjected to hindlimb suspension. D) Ileum from a mouse treated with hindlimb suspension and irradiated with 2 Gy of protons. Original magnifications 200×. E) Quantitation of Claudin-3 breaks in terminal ileum columnar epithelium. The number of breaks or interruptions in claudin-3 staining per 1,000 enterocytes for each animal per treatment were averaged. The number of goblet cells per 1,000 enterocytes is given as a control. (a) SD, standard deviation for breaks in claudin-3 staining, within animals in a treatment. (b) p-values for breaks in claudin-3 staining determined by ANOVA analyses with post-hoc group comparisons using Bonferroni correction, F = 4.57, p = 0.008. (c) ns, not significant. Tissue from 10 animals was analyzed for each condition.

To determine mechanisms of increased LPS translocation, immunohistochemical staining for the tight junction protein Claudin-3 was performed on terminal ileum 4 days after irradiation and/or 6 days after hindlimb unloading. A significant increase in the number of breaks and reductions in staining were observed in 2 Gy proton or gamma radiation treated and hindlimb suspended animals (Figure 5). The frequency of breaks was not significantly increased in ileum from radiation treated or suspended animals compared to untreated.

**Figure 6:**
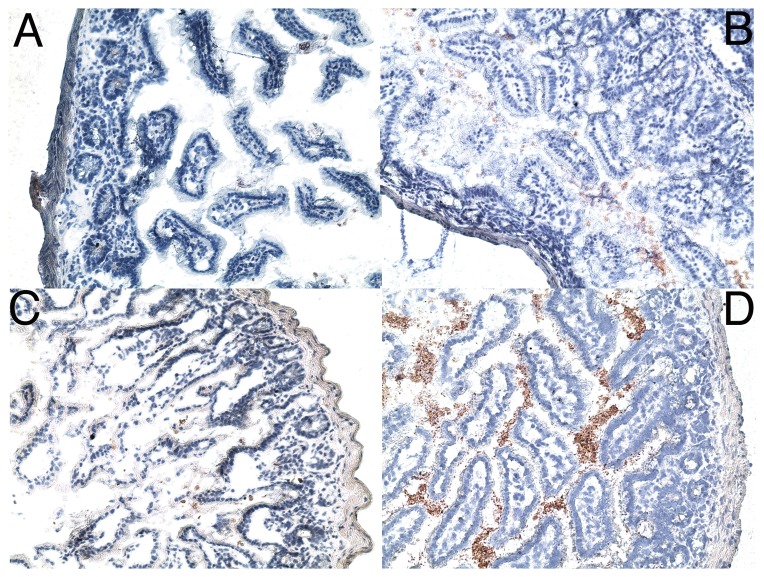
SPE-like radiation and hindlimb suspension induces the accumulation of LPS in subepithelial regions of the ileum. Terminal ileum obtained 4 days post irradiation and/or 6 days post hindlimb suspension or from control animals was stained for LPS using a mouse mAb specific for E. coli LPS. A) Ileum from a control treated mouse. B) Ileum from a mouse irradiated with 2 Gy of 70 MeV protons. C) Ileum from a mouse subjected to hindlimb suspension. D) Ileum from a mouse treated with hindlimb suspension and irradiated with 2 Gy of protons. Original magnifications 200×. Tissue from 10 animals was analyzed for each condition.

The presence of an increased frequency of breaks in the epithelial cell barrier of the GI tract is suggestive that it is mechanistically responsible for the increase in translocation of bacterial products. To strengthen this causal association, terminal ileum had its lumen extensively flushed with saline before mounting and was stained with Abs that recognize LPS, a mouse mAb against E. coli (J5) LPS or a goat anti-lipid A IgG that cross-reacts with *Pseudomonas aeruginosa*, *Klebsiella pneumoniae*, *E. coli O157*, *Salmonella enteriditis*, *Enterobacter aerogenes*, *E. hermanii*, *Yersinia enterocolitica*, and *Shigella sonnei*. Control animals demonstrated no LPS specific staining in the intervillous space (Figure 6A), while very low levels of staining were observed in animals treated with 2 Gy of proton radiation or hindlimb unloading alone (Figure 6B and C). High level diffuse staining was observed in animals treated with both proton radiation and hindlimb unloading (Figure 6D). Similar findings were found for gamma radiation treated mice.

## Discussion

Future space travel will include extended stays outside of the Earth's magnetic field, which offers protection from solar radiation. Thus, in addition to factors associated with space exploration circling the Earth that can impair immune function, including altered nutritional intake [4], situational and confinement stress [1], disturbance of circadian rhythms [3], and reduced weight bearing (reviewed in [2]), the effect of radiation from SPEs must also be considered. Astronauts could receive up to 2 Gy of radiation to the bone marrow [31] and up to ∼32 Gy to the skin [32] during a strong SPE. Using models of spaceflight that encompass most of the described stresses likely to be encountered during space travel and adding radiation that models the energy spectrum of an SPE, we demonstrate a rapid alteration in immunologic status with systemic immune activation and demonstrate that this is mediated by an increase in circulating bacterial LPS, which is caused by a breakdown in the ability of the GI tract to segregate LPS from underlying tissues.

We previously demonstrated that 2 Gy of proton or reference gamma radiation led to a transient increase in immune activation associated with an increase in circulating LPS [33]. In this report, we extend these findings to demonstrate that in the optimal land based model of reduced weight bearing, hindlimb suspension, there is a synergistic effect when mice are additionally exposed to SPE-like radiation. Interestingly, hindlimb suspension alone led to an increase in circulating LPS that decreased with increasing amounts of time in suspension (Figure 1B). This may reflect contributions from various elements of microgravity modeling, including fluid shifts, situational and confinement stress, altered nutritional intake, and changes in circadian rhythms. The addition of SPE-like radiation to hindlimb suspension both increased circulating LPS and extended the duration of the elevated LPS to 4 days post irradiation (Figure 1), while radiation alone resulted in circulating LPS levels returning to baseline by 2 days post irradiation. Associated markers of immune activation, LBP and sCD14, were also elevated at 24 hr and remained elevated 4 days after irradiation when mice were also hindlimb unloaded. We suggest synergy versus additive, as the reported half-life of LPS delivered as a large dose (1 mg/kg) was calculated to be approximately 12 hr with a peak circulating level of approximately 360 ng/ml [44]. Using a conversion of 100 pg of US standard endotoxin EC-5 equals 1 EU, these mice had approximately 3,600 EU/ml of LPS. Human studies of low level transient increases in LPS demonstrated much faster clearance [45]. Even with a 12 hr half-life, the increased level of LPS observed after 2 Gy of radiation and hindlimb suspension would not be measurable 4 days later, demonstrating that radiation and suspension result in a stronger and much prolonged impairment to the containment of bacterial products with resultant immune activation. This was confirmed by the demonstration of continued tight junction interruptions and the presence of LPS in the intervillous space in the terminal ileum. The observation that LPS was present across the epithelial barrier is strong evidence directly supporting a mechanism where breakdown of the epithelium leads to traversal of bacterial products and systemic circulation. Whether radiation or suspension has additional effects that alter the ability of the GI tract epithelial membrane to contain bacterial products other than reducing tight junctions could not be determined.

The ability of radiation to induce inflammation and augment other sources of inflammation has been described in other systems. Five Gy of total body irradiation of mice led to a transient increase in circulating LPS with systemic immune activation [46]. In a model of radiation with burn injury, the combination of 5 Gy of gamma radiation with full-thickness scald injury led to an increase in the duration of circulating IL-6 and an increase level and duration of circulating TNF-α [47], which is in agreement with other studies [48]. Although, in a study of rats given 12 Gy of gamma radiation and 30% total body surface third-degree burns, the addition of radiation led to no increase in circulating IL-6 or TNF-α levels compared to burns alone [49]. In a model of skin wounding with gamma radiation, the combination led to increased levels of circulating proinflammatory cytokines and chemokines, and interestingly, skin wounding led to a further increase in radiation induced changes to ileal morphology, including a reduction in villous width and a reduction in the thickness of the tunica muscularis [50], although findings in different systems obtained contrary results [51]. The addition of 12 or 24 Gy of electron radiation to wounding in rats resulted in impaired healing and elevated local levels of TNF-α and IFN-γ [52]. These studies in different systems of inflammatory insult, in part, demonstrate a similar combinatorial effect with radiation leading to elevated levels of immune activation in support of our findings.

A major difficulty of studying immunologic dysfunction during space travel involves the model systems needed to perform the analyses on Earth. The hindlimb unloading model is the accepted best system and has been directly compared to animals subjected to spaceflight [35], [36]. A review of the data suggests that the hindlimb unloading model produces many of the alterations in immune function found in space flight, and in particular finds that changes in dynamic immune responses are reproduced (reviewed in [36], [53]). In addition, the hindlimb unloading model replicates a number of aspects of spaceflight in addition to weightlessness, including situational and confinement stress, altered circadian rhythms, and altered nutritional intake [36], [54], [55], [56]. The immune alterations observed with hindlimb unloading decreased over the length of time the mice were suspended, suggesting that they adapted to the effects or stress of hindlimb unloading.

Numerous alterations to immune function have been observed after short or long space missions in humans and animals and in animals during land-based models of spaceflight (review in [17], [57], [58], [59]). Consistent observations include altered distributions of immune cells and variations in cytokines released in response to stimulations. This includes an increase in anti-inflammatory cytokines and a decrease in TNF-α in LPS stimulated spleen cells [60], reductions in interferon-γ and IL-2 following PMA and ionomycin stimulation of peripheral blood cells of astronauts [15], and reduced NK cell number and functionality [13], [14]. These alterations to immune function are very similar to those observed in settings of increased immune activation caused by exposure to pathogen associated molecular patterns (PAMPs), such as LPS [61], [62], [63], [64], [65], [66]. Specific examples include; a reduction in proinflammatory cytokine production by myeloid cells [66], [67], a reduction in antigen-specific T cell effector cytokine responses [61], and a reduction in circulating NK cells [68].

The alterations in responses mediated by exposure to PAMPs is classically called “tolerance”. It is characterized by a state in which subsequent responses are diminished in quality and quantity, potentially to protect the host by limiting excessive inflammation and preventing septic shock [66], [69]. Continuous or extended exposure, as has been observed in multiple disease states [43], [62], [63], [70], [71], [72], [73], can lead to long-lived immune dysfunction. Of particular interest, LBP, a common marker of immune activation, has been observed to be elevated in astronauts' plasma [17], [67]. Future studies are needed to determine whether a causal relationship exists that links our findings in models of spaceflight and SPE radiation exposure to immune dysfunctions observed during and after extended spaceflight and solar radiation exposure. Ongoing studies are determining the clinical effect of radiation and hindlimb suspension in various challenge models to quantitate the level of immune dysfunction and its clinical significance.

Studies examining potential countermeasures that either reverse or mitigate the effects of radiation and hindlimb suspension on the GI tract or on the immune system are ongoing. Extended duration missions outside of the protection of the Earth's magnetic field that will include extravehicular activities will need to be prepared for such encounters, as current technology cannot forecast the risk of exposure to SPE radiation. Therapies that can be studied include antibiotics for infections due to either translocated bacteria and/or immunosuppression, granulocyte colony-stimulating factors to increase innate immune activity, as well as future approaches using cytokines to stimulate GI tract epithelial cell repair and potentially host stem cell mobilization.

Future missions in space will include extended durations outside of the Earth's magnetic field and its protection from solar and galactic radiation. Numerous studies of the effect of conditions encountered during space travel in land-based models or during space travel in animals and humans have descriptively identified numerous immune alterations, many of which are shared between actual spaceflight and land-based models. In this report, we identify a common mechanism whereby SPE-like radiation and land-based models of space travel induce systemic immune dysfunction. Future studies will determine the contribution this mechanism has on the various immune deficiencies identified in models or actual spaceflight. A major concern in the planning of space missions is the demand for effective countermeasures for spaceflight and SPE-mediated impairments to physiologic functions. This is especially important for extended missions, in which the only treatments available are those brought with the mission. The development of countermeasures is improved by the identification of both the descriptive nature of the dysfunction, but most importantly, by the mechanisms responsible for the dysfunction.
